# Supplementary material for: Estimating total human deaths from rabies: A statistical framework leveraging reports of rabies transmission from deceased organ and tissue donors
Source: PLoS Negl Trop Dis. 2026 Sep 21;20(9):e0014731. doi: 10.1371/journal.pntd.0014731 (PMC13592719; doi:10.1371/journal.pntd.0014731)
Supplement: Supplementary file 6 [file pntd.0014731.s005.zip › Result_B/Kazakhstan/Figure_S3_Kazakhstan.pdf]

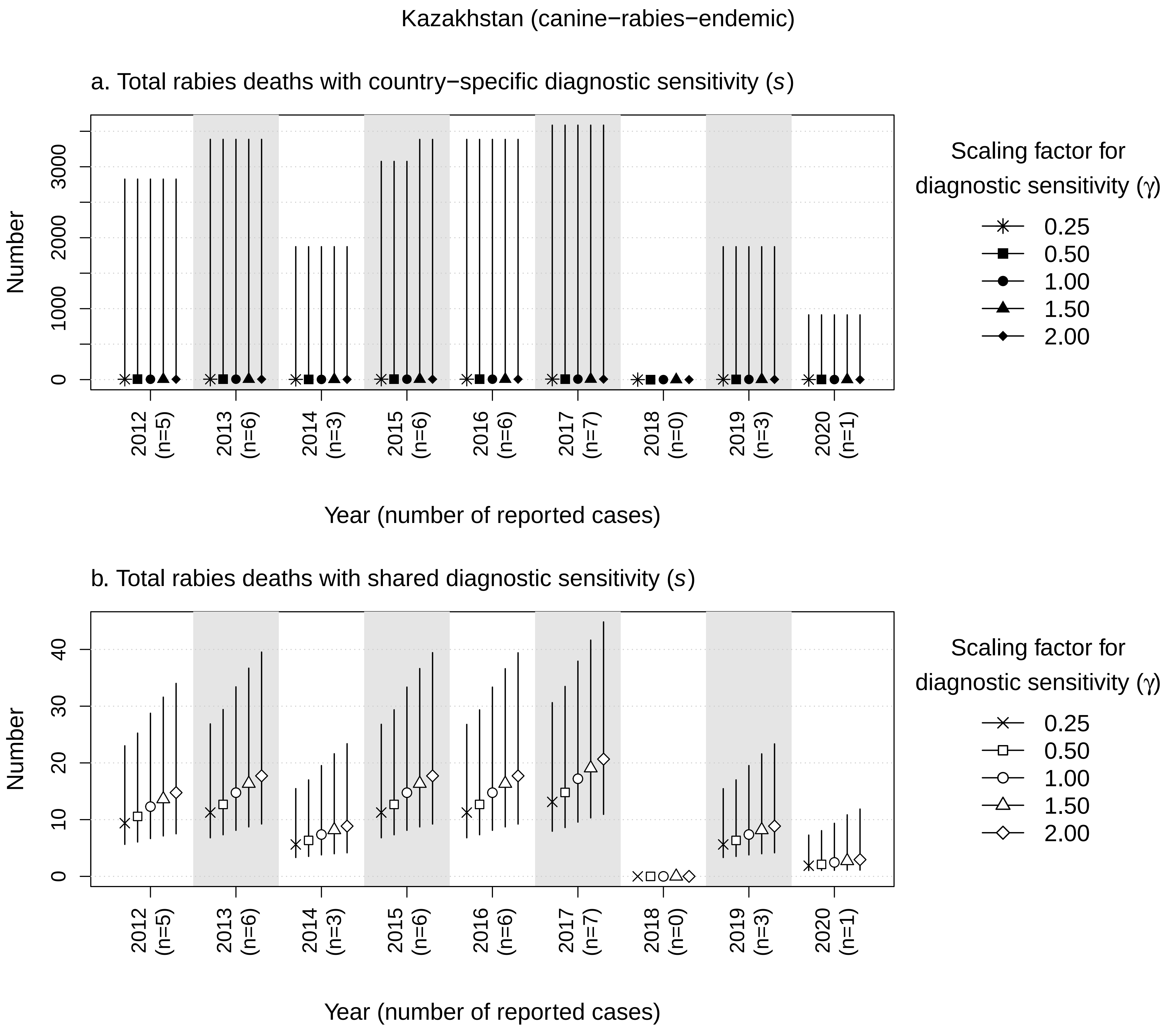
Kazakhstan (canine−rabies−endemic)
a. Total rabies deaths with country−specific diagnostic sensitivity (s)
Number
0
1000
2000
3000
2012 (n=5)
2013 (n=6)
2014 (n=3)
2015 (n=6)
2016 (n=6)
2017 (n=7)
2018 (n=0)
2019 (n=3)
2020 (n=1)
Year (number of reported cases)
Scaling factor for diagnostic sensitivity (γ)
0.25
0.50
1.00
1.50
2.00
b. Total rabies deaths with shared diagnostic sensitivity (s)
Number
0
10
20
30
40
2012 (n=5)
2013 (n=6)
2014 (n=3)
2015 (n=6)
2016 (n=6)
2017 (n=7)
2018 (n=0)
2019 (n=3)
2020 (n=1)
Year (number of reported cases)
Scaling factor for diagnostic sensitivity (γ)
0.25
0.50
1.00
1.50
2.00
